# Ascending Aortic Graft Anastomotic Thrombosis Mimicking Prosthetic Graft Endocarditis

**DOI:** 10.7759/cureus.106292

**Published:** 2026-04-01

**Authors:** Juan O Rodriguez Padilla, Juan C Rivera-Martinez, Camila Villacreses, Felix Carrillo, Omar Nass

**Affiliations:** 1 Internal Medicine, Lakeland Regional Health, Lakeland, USA; 2 Hospital Medicine, Lakeland Regional Health, Lakeland, USA; 3 Cardiology, Lakeland Regional Health, Lakeland, USA

**Keywords:** anastomotic thrombosis, ascending aortic graft, culture negative endocarditis, mechanical aortic valve, multimodality imaging, prosthetic graft endocarditis, prosthetic graft thrombosis, transesophageal echocardiography

## Abstract

Thrombosis at an ascending aortic graft anastomosis is uncommon and can mimic prosthetic graft endocarditis. A 42-year-old male with bicuspid aortic valve disease status post mechanical aortic valve replacement and ascending aortic graft repair for aneurysmal disease presented for evaluation of recurrent arterial thromboembolic events and concern for prosthetic material complications. Transesophageal echocardiography demonstrated a highly mobile mass at the distal graft anastomosis, while the mechanical valve appeared structurally normal with no vegetation. Computed tomography angiography localized an irregular intraluminal lesion to the distal anastomotic suture line. Surgical exploration revealed an organized, calcified mass that was resected. Blood cultures obtained before antibiotics remained negative, and repeat blood cultures were also negative. Intraoperative tissue cultures, including bacterial, fungal, and acid-fast cultures, were negative, and histopathology showed an organized thrombus with dystrophic calcification and inflammation without microorganisms on special stains. Targeted serological testing and tissue molecular testing were not performed. The multidisciplinary review concluded that the findings were most consistent with anastomotic thrombosis rather than active prosthetic graft endocarditis, and empiric antibiotics were discontinued; however, culture-negative endocarditis was not fully excluded because targeted serological and molecular testing were not performed, and the sterile designation therefore remains presumptive.

## Introduction

Ascending aortic graft complications after cardiac surgery can be morbid and diagnostically challenging. Anastomotic thrombosis and prosthetic graft endocarditis can appear similar because both may present with a mobile mass on prosthetic material, systemic thromboembolism, and constitutional symptoms. However, management differs substantially: prosthetic graft endocarditis typically requires prolonged antimicrobial therapy and may require reoperation, whereas anastomotic thrombosis is managed with anticoagulation optimization and, in selected cases, surgical excision to reduce embolic risk [1,2].

For readers outside cardiology and infectious disease, bicuspid aortic valve disease is a relevant background because it is frequently associated with aortopathy and commonly leads to prior aortic valve surgery and ascending aortic repair. In that context, subsequent prosthetic graft complications may present with nonspecific constitutional symptoms, embolic events, or an incidentally detected mobile mass on imaging. When such a mass is identified in a patient with prior prosthetic cardiac material, distinguishing thrombus from active infection becomes a clinically important diagnostic challenge because the therapeutic and prognostic implications differ substantially.

Most diagnostic frameworks for infective endocarditis were developed for valvular infection and may be less informative when suspected pathology is confined to prosthetic aortic graft material rather than the valve itself [1,2]. In addition, culture-negative endocarditis complicates interpretation because negative blood cultures do not by themselves exclude infection in patients with prosthetic cardiovascular material; the proportion of endocarditis cases without microbiologic confirmation varies by clinical context, patient population, and diagnostic resources available, and this figure should be interpreted cautiously in any individual case [3,4]. We therefore report a case in which multimodality imaging, operative findings, microbiology, and histopathology were integrated to distinguish graft anastomotic thrombosis from prosthetic graft endocarditis, while acknowledging that culture-negative endocarditis could not be fully excluded in the absence of targeted serological and molecular testing.

## Case presentation

A 42-year-old male with a past medical history significant for bicuspid congenital aortic valve stenosis underwent his first cardiac operation in early childhood; however, the exact nature of that procedure could not be confirmed from the records currently available for this report. He later underwent mechanical aortic valve replacement with ascending aortic repair for an ascending aortic aneurysm in 2024. He was maintained on warfarin for the anticoagulation of his mechanical valve. Although he reported adherence and outpatient monitoring, historical international normalized ratio values were variable, reportedly ranging from as low as 1.0 to as high as 4.0.

In late 2025, he developed a right middle cerebral artery ischemic stroke. Around the same period, he also experienced a left brachial artery occlusion requiring brachial artery stent placement. He was followed as an outpatient with plans for evaluation of possible hypercoagulability given recurrent arterial thromboembolic events; however, detailed results of any outpatient hypercoagulable evaluation were not available in the records accessible for this report. Several weeks after routine dental work, he developed a self-limited episode of fever and chills that resolved over several days without antibiotics. He subsequently presented for elective transesophageal echocardiography to evaluate for prosthetic cardiovascular material complications in the setting of recurrent thromboembolic events.

On admission, vital signs were within normal limits. Cardiovascular examination revealed a normal first heart sound and a mechanical second heart sound click without murmurs or rubs. Lung auscultation was clear bilaterally. There was no jugular venous distension, peripheral edema, or peripheral stigmata of endocarditis. Initial laboratory testing demonstrated preserved renal function with normal blood urea nitrogen and creatinine. Coagulation testing showed a therapeutic international normalized ratio, slightly prolonged prothrombin time, and mildly prolonged activated partial thromboplastin time (Table 1). The mildly prolonged activated partial thromboplastin time was noted descriptively and was not used as a basis for the final diagnostic conclusion. Blood cultures were obtained prior to antibiotic administration. Four sets of blood cultures were obtained: two sets on admission and two sets subsequently, and all remained negative, with no growth through day five. Repeat blood cultures obtained on follow-up were also negative. Despite cultures being obtained prior to antibiotics, he received empiric vancomycin and piperacillin-tazobactam in the emergency department.

**Table 1:** Admission Laboratory Parameters

Laboratory Parameter	Value	Reference Range	Interpretation
Blood Urea Nitrogen	12 mg/dL	7 to 20 mg/dL	Normal
Creatinine	0.71 mg/dL	0.7 to 1.3 mg/dL	Normal
International Normalized Ratio	2.6	0.9 to 1.1 (therapeutic range for mechanical valve: 2.5 to 3.5)	Therapeutic
Prothrombin Time	30 seconds	11 to 13.5 seconds	Prolonged
Activated Partial Thromboplastin Time	42.8 seconds	25 to 35 seconds	Mildly prolonged

**Figure 1:**
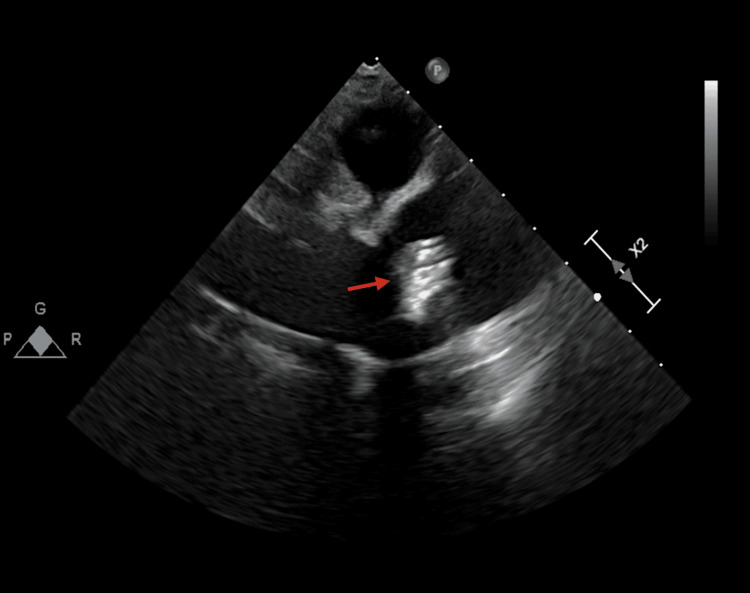
Transesophageal Echocardiography Image Showing the Mobile Mass Transesophageal echocardiography image demonstrating a pedunculated, mobile echogenic mass at the distal anastomotic region of the ascending aortic graft (red arrow). The mechanical aortic valve prosthesis is visualized and appears well seated.

Transesophageal echocardiography demonstrated preserved biventricular systolic function with a left ventricular ejection fraction greater than 55%. The mechanical aortic valve was well seated and functioned normally with appropriate leaflet excursion, trivial aortic insufficiency, and no evidence of vegetation, dehiscence, periprosthetic leak, or other structural abnormality. A pedunculated, highly mobile, echogenic mass measuring approximately 12 millimeters was identified at the distal anastomotic region of the ascending aortic graft, raising concern for a thrombus versus a vegetation (Figure 1, Video 1). The mass moved independently from the mechanical valve throughout the cardiac cycle, and the consistently normal appearance of the valve redirected diagnostic attention toward a graft anastomotic process rather than prosthetic valve endocarditis.

**Video 1:** Transesophageal Echocardiography Clip Demonstrating Mobile Anastomotic Mass Transesophageal echocardiography clip demonstrating independent motion of a highly mobile mass at the distal anastomotic region of the ascending aortic graft throughout the cardiac cycle, with normal mechanical aortic valve structure and function.

**Figure 2:**
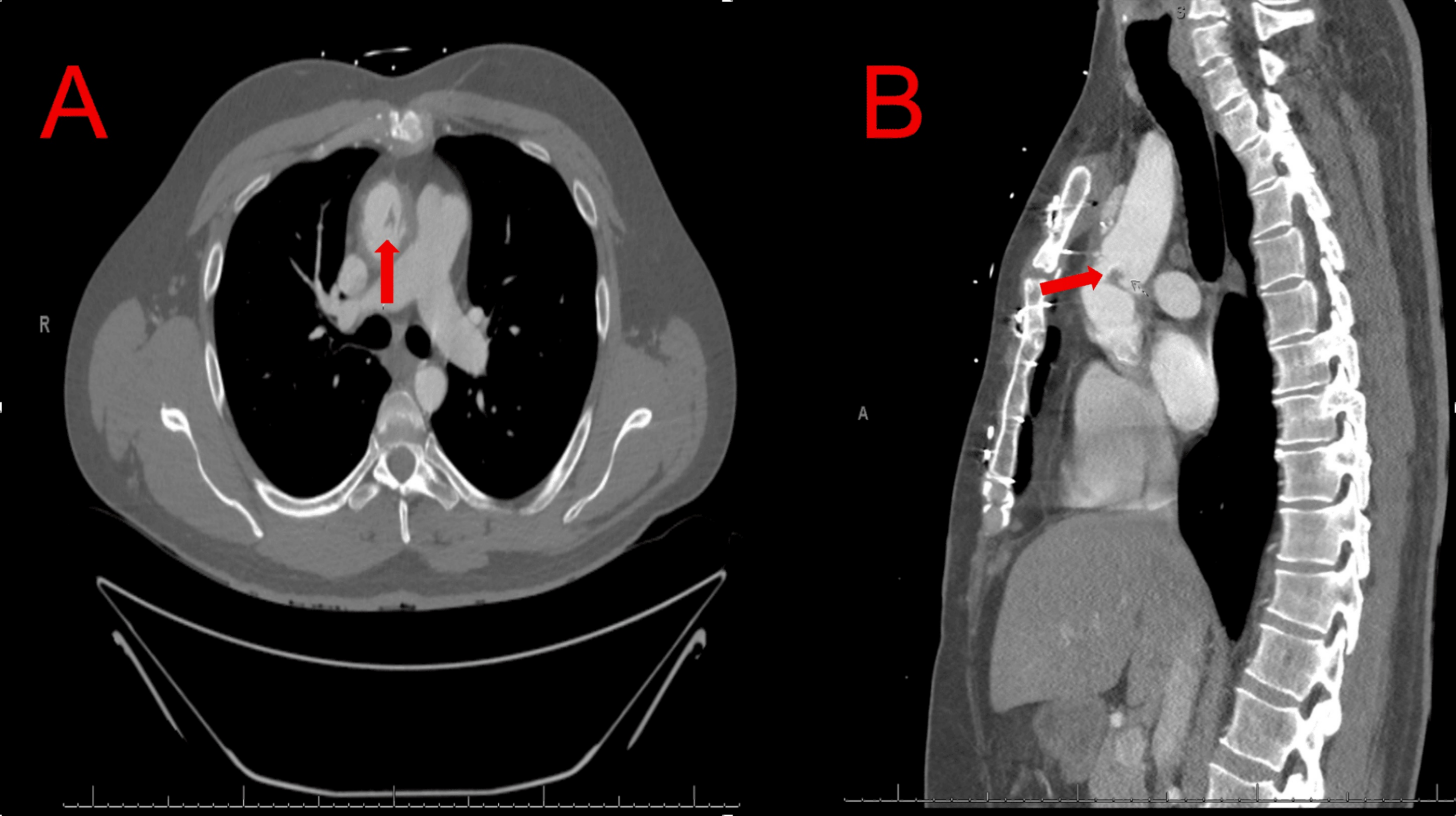
Computed Tomography Angiography Demonstrating an Intraluminal Lesion at the Ascending Aortic Graft Computed tomography angiography of the chest with intravenous contrast demonstrating an intraluminal lesion at the ascending aortic graft. Panel A (axial view) shows a focal irregular hypodense lesion projecting into the lumen of the ascending aortic graft (arrow). Panel B (sagittal reconstruction) localizes the lesion to the distal anastomotic site at the junction of the graft and native aorta (arrow). No periaortic fluid collection, pseudoaneurysm, or aortic dissection is identified.

Computed tomography angiography of the chest with intravenous contrast was performed for further characterization (Figure 2). Axial imaging demonstrated a focal, irregular hypodense lesion projecting into the lumen along the left anterolateral wall of the aortic graft. Sagittal reconstruction localized the lesion to the distal anastomotic site at the junction of the graft and native aorta, supporting a focal anastomotic rather than valvular process. There was no evidence of aortic dissection, periaortic fluid collection, or pseudoaneurysm. Given recurrent thromboembolic events, an antecedent self-limited febrile episode after dental work, a mobile mass at the graft anastomosis on multimodality imaging, and negative blood cultures, the leading considerations were prosthetic graft endocarditis versus anastomotic thrombosis.

Anticoagulation was maintained with continuous intravenous heparin infusion. Tirofiban was continued because of the recent left brachial artery stent placed for an enlarging pseudoaneurysm. Review of the available records identified the stent as a Gore Viabahn endoprosthesis measuring 6 mm × 10 cm. However, the intended duration of tirofiban therapy, the specific service or clinician directing this decision, and the details of the perioperative antithrombotic risk assessment discussion were not retrievable from the records currently available for this report. Cardiothoracic surgery was consulted for operative management. On hospital day 4, he underwent surgical exploration with resection of the distal ascending aortic graft and reconstruction using right common femoral artery cannulation for cardiopulmonary bypass. Intraoperative transesophageal echocardiography again demonstrated the mobile mass at the distal anastomosis and confirmed normal structure and function of the mechanical aortic valve without vegetation. Operative inspection revealed circumferential ridge formation at the distal anastomotic suture line with calcific excrescences corresponding to the preoperative imaging findings. The lesion appeared organized and firm with a pale appearance rather than friable or purulent. The redundant internal graft portion containing the mass was resected, and the graft was reconstructed by closing against the distal anastomotic cuff. Tissue was sent for microbiologic cultures and histopathologic analysis. Postprocedure transesophageal echocardiography confirmed complete removal of the mass and normal function of the mechanical aortic valve.

**Figure 3:**
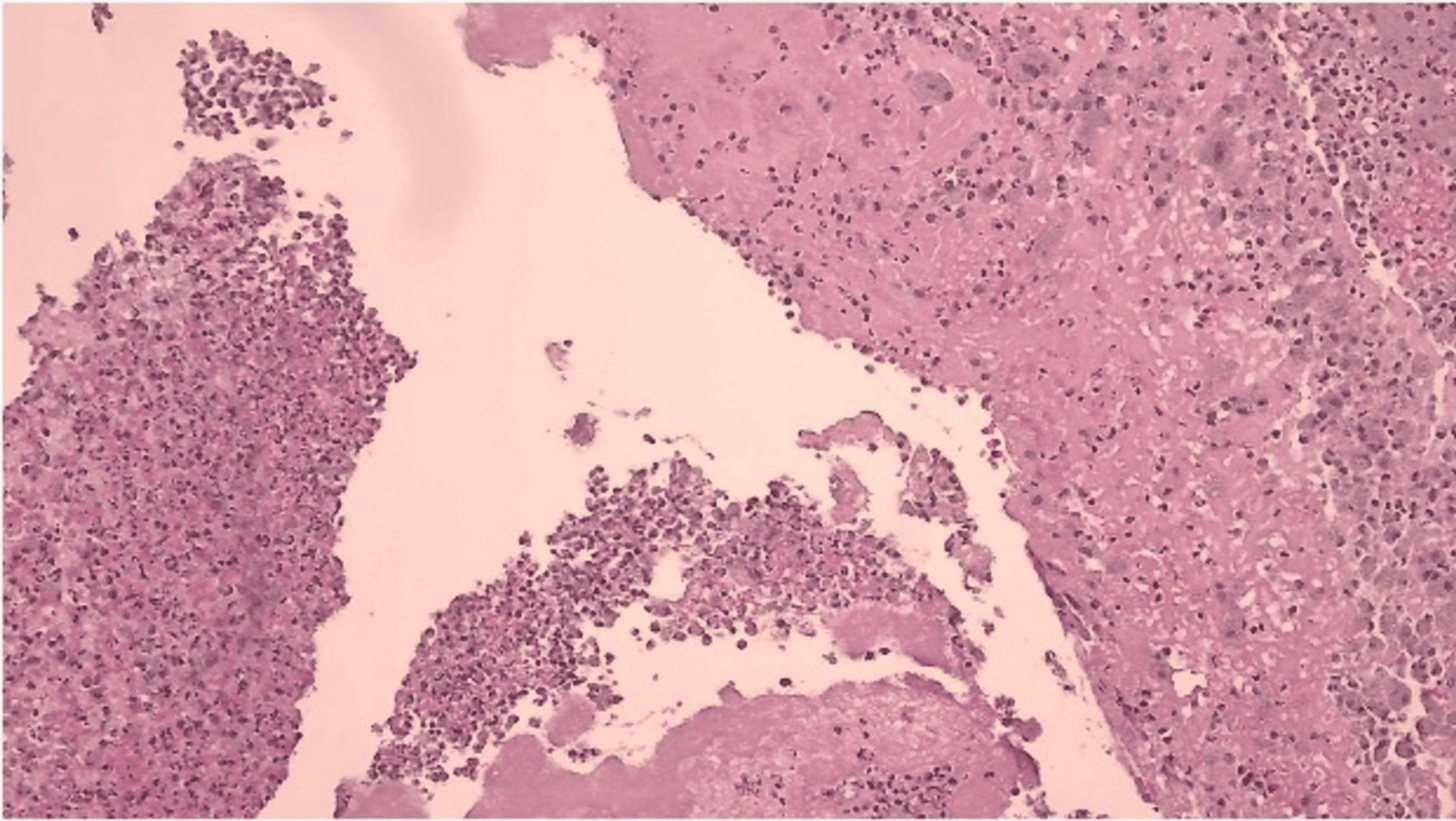
Histopathology of Excised Anastomotic Tissue (Low Power) Hematoxylin and eosin staining at low power demonstrates fragments of organized thrombus with associated inflammatory infiltrate. No microorganisms were identified on histologic evaluation. Exact magnification was not available from the source pathology records and has not been added.

**Figure 4:**
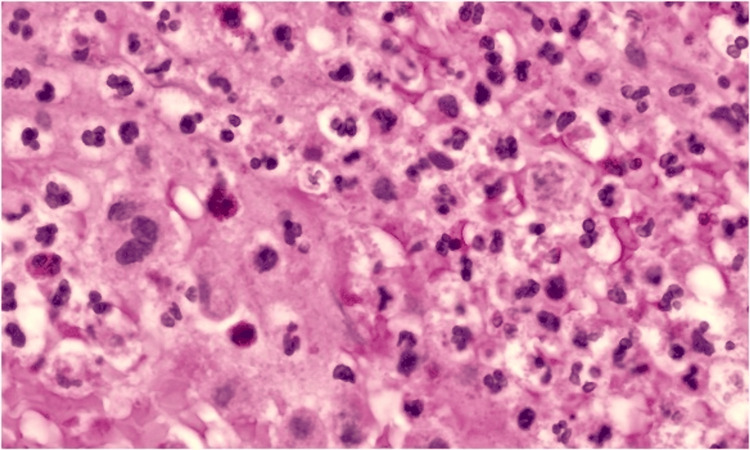
Histopathology of Excised Anastomotic Tissue (High Power) Hematoxylin and eosin staining at higher magnification demonstrates fibrinous material with mixed inflammatory cells, including neutrophils and foamy histiocytes. Special stains, including Gram stain and Gomori methenamine silver stain, were negative for microorganisms. Exact magnification was not available from the source pathology records and has not been added.

Intraoperative tissue cultures, including bacterial, fungal, and acid-fast cultures, demonstrated no growth. Surgical pathology demonstrated fragmented, organized thrombus with dystrophic calcification and an associated inflammatory reaction on hematoxylin and eosin staining (Figures 3, 4). On higher magnification, fibrinous material with mixed inflammatory cells, including neutrophils and foamy histiocytes, was noted. Special stains, including Gram stain and Gomori methenamine silver stain, were negative for microorganisms. No organisms were identified. Targeted serological testing and tissue molecular testing, including polymerase chain reaction, were not performed.

Postoperatively, he recovered without recurrent constitutional symptoms or hemodynamic instability but required transfusion for anemia related to surgical blood loss. Anticoagulation was resumed with intravenous heparin bridging to warfarin with an international normalized ratio goal of 2.5 to 3.5 given the mechanical valve and history of thromboembolic events [5,6]. Because of persistent concern for possible culture-negative graft endocarditis in the setting of antecedent dental work and transient fever, the infectious diseases service recommended empiric intravenous ceftriaxone and daptomycin for a planned six-week course. A peripherally inserted central catheter was placed on hospital day 5 to facilitate outpatient therapy. He was discharged on hospital day 6 with plans for completion of intravenous antibiotics, cardiology follow-up, and international normalized ratio monitoring.

Within 48 hours, he returned with erythema, warmth, swelling, and pain at the catheter insertion site. Venous duplex ultrasonography demonstrated superficial vein thrombosis at the catheter site without deep venous thrombosis, and the catheter was removed. On hospital day 8, a multidisciplinary case review involving cardiology, cardiothoracic surgery, and infectious diseases was convened (Table 2). The group reviewed persistently negative blood cultures obtained prior to antibiotics; negative intraoperative tissue cultures, including bacterial, fungal, and acid-fast cultures; intraoperative gross appearance consistent with organized thrombus; histopathology demonstrating thrombus with calcification and inflammation without organisms on multiple stains; absence of ongoing fever or systemic inflammatory response after surgical excision; and clinical improvement after source control. The consensus was that the aggregate findings were most consistent with anastomotic thrombosis with secondary inflammation rather than active prosthetic graft endocarditis; the group acknowledged that culture-negative prosthetic graft infection could not be fully excluded because targeted serological and molecular testing had not been performed, and antibiotics were discontinued on that basis. He was discharged on hospital day 9 on warfarin with an international normalized ratio goal of 2.5 to 3.5 and follow-up arranged with cardiology, cardiothoracic surgery, and primary care.

**Table 2:** Clinical Course and Key Findings During Hospitalization CTA, computed tomography angiography; INR, international normalized ratio; PICC, peripherally inserted central catheter; TEE, transesophageal echocardiography.

Hospital Day	Clinical Events	Imaging and Procedures	Microbiology	Management Decisions
1 (Admission)	Presentation for elective TEE; prior transient fever after dental work	TEE: mobile approximately 12 mm mass at distal graft anastomosis; mechanical valve normal (Figure 1, Video 1)	Blood cultures obtained prior to antibiotics; no growth through day 5	Empiric vancomycin and piperacillin-tazobactam in the emergency department; anticoagulation maintained; surgical consultation
2 to 3	Clinical stability; surgical planning	CTA: irregular lesion localized to distal anastomosis; no dissection, fluid collection, or pseudoaneurysm (Figure 2)	Blood cultures negative	Continued anticoagulation; operative management planned
4	Surgical exploration and graft reconstruction	Intraoperative TEE confirmed anastomotic mass and normal valve; organized calcified lesion resected	Tissue cultures obtained (bacterial, fungal, acid-fast)	Excision and graft reconstruction; tissue sent for pathology
5	Postoperative recovery	Pathology: organized thrombus with calcification; stains negative (Figures 3, 4)	Blood and tissue cultures negative	Empiric ceftriaxone and daptomycin planned; PICC placed
6	Discharge	Stable	Cultures negative	Discharged with intravenous antibiotics and a follow-up plan
7 to 8	Readmission for catheter site symptoms	Duplex ultrasonography: superficial thrombosis at the catheter site	Repeat blood cultures negative (approximately two weeks later)	Catheter removed; multidisciplinary review; antibiotics discontinued
9	Final discharge	Stable, afebrile	All cultures negative	Warfarin with INR goal 2.5 to 3.5; outpatient follow-up

## Discussion

This case highlights a diagnostic dilemma in which a mobile mass on prosthetic aortic graft material, recurrent arterial thromboembolism, and a preceding self-limited febrile episode created substantial concern for prosthetic graft endocarditis. The key discriminator in this patient was anatomic localization: transesophageal echocardiography repeatedly demonstrated a normally functioning mechanical aortic valve without vegetation or perivalvular complication while identifying a discrete, highly mobile mass at the distal graft anastomosis [1]. Computed tomography angiography provided complementary localization by confirming that the lesion arose from the distal anastomotic suture line and by excluding findings that would heighten suspicion for infection, such as periaortic fluid collection or pseudoaneurysm [1,2].

Although infective endocarditis diagnostic frameworks, such as the modified Duke and European Society of Cardiology criteria, provide important clinical context, they were not fully determinative in this case because the suspected pathology was confined to prosthetic graft material rather than the valve itself. These criteria were developed primarily with valvular infection in mind, and their performance may differ when the diagnostic question involves graft-confined pathology [1,2]. Based on the documented findings available in this report, the case did not satisfy the criteria for definite infective endocarditis. Even so, empiric antibiotic therapy was clinically reasonable early in the evaluation, given the combination of embolic stroke, antecedent febrile symptoms, prosthetic cardiac material, and a mobile mass on imaging. Diagnosis in this setting required integration of multimodality imaging, operative inspection, microbiology, histopathology, and multidisciplinary review rather than reliance on a valve-centered framework alone.

Despite negative blood cultures, the possibility of prosthetic material infection remained clinically important because culture-negative endocarditis occurs in a meaningful minority of cases and because missing a prosthetic infection carries high morbidity [3,4]. In this case, targeted serological testing and tissue molecular testing were not performed, and empiric antibiotics were initially pursued after culture acquisition, given the clinical uncertainty and antecedent symptoms. However, definitive clarification required operative assessment and tissue diagnosis. Intraoperatively, the lesion appeared organized and firm with calcific excrescences rather than friable or purulent [7]. Histopathology showed organized thrombus with dystrophic calcification and an inflammatory reaction without organisms on special stains [8]. In addition, blood cultures obtained before antibiotics and repeated subsequently remained negative, and intraoperative tissue cultures, including bacterial, fungal, and acid-fast cultures, showed no growth [3,4]. Taken together, these findings were most consistent with anastomotic thrombosis rather than active graft infection; however, because targeted serological and molecular testing for culture-negative endocarditis organisms was not performed, culture-negative prosthetic graft infection could not be fully excluded, and the sterile designation should be regarded as presumptive rather than definitive.

The pathophysiology of anastomotic thrombosis is directly relevant to this case and provides a mechanistic explanation for thrombus formation at the graft to native vessel junction despite therapeutic systemic anticoagulation [7,8]. At this anatomic site, multiple prothrombotic factors converge: endothelial disruption from surgical manipulation and suture placement; exposure of the subendothelial collagen matrix and thrombogenic graft material; compliance mismatch between the rigid synthetic graft and elastic native aortic wall; and flow disturbances, including turbulence, stasis, and recirculation zones at regions of abrupt geometric transition [7,8]. Compliance mismatch is particularly important in the pathogenesis of anastomotic complications; the nonelastic nature of surgical suture material creates a focal reduction in vessel compliance at the suture line, with paradoxically hypercompliant zones immediately adjacent to this rigid segment where intimal hyperplasia and thrombus may preferentially develop in response to repetitive mechanical stress during the cardiac cycle [7-9]. Over time, organized thrombus undergoes progressive structural remodeling, including calcification, fibrosis, and the development of surface irregularities that increase both embolic potential through fragmentation and echocardiographic resemblance to vegetation through acoustic heterogeneity [10].

The patient's course also illustrates the importance of reassessing empiric antimicrobial therapy as additional data accumulate. Although empiric therapy can be reasonable early in evaluation when prosthetic infection is a concern, prolonged intravenous therapy carries meaningful risks, including line-associated complications, as occurred here [11]. A multidisciplinary review integrating imaging localization, operative appearance, microbiology, histopathology, and clinical trajectory supported discontinuation of antibiotics and refocused management on long-term antithrombotic strategy [1-4].

This case also raises important considerations for antithrombotic management in a patient with a mechanical valve and recurrent thromboembolism. Although the admission international normalized ratio was therapeutic, historical values reportedly ranged from 1.0 to 4.0, and recurrent arterial events had prompted outpatient evaluation for possible hypercoagulability [5,6]. Tirofiban was continued during admission because of the recent left brachial artery Gore Viabahn endoprosthesis placed for an enlarging pseudoaneurysm, although the intended duration of therapy, the specific service or clinician directing this decision, and the details of the perioperative bleeding risk assessment were not recoverable from the currently available records. Current guidelines support a higher-intensity vitamin K antagonist strategy, with an international normalized ratio goal of 2.5 to 3.5, for mechanical aortic valve patients with prior thromboembolism or other risk factors [5,6]. Close monitoring and evaluation for contributing prothrombotic conditions are appropriate [12].

Limitations include the absence of targeted serological testing and tissue polymerase chain reaction for culture-negative endocarditis organisms, incomplete availability of prior operative and outpatient hypercoagulability records, and limited long-term follow-up data within this report to assess for recurrence after surgical excision and anticoagulation optimization [3,4].

## Conclusions

Ascending aortic graft anastomotic thrombosis can closely mimic prosthetic graft endocarditis when a mobile mass is detected on prosthetic material in a patient with thromboembolic events and antecedent constitutional symptoms. Accurate diagnosis depends on precise anatomic localization showing a normal mechanical aortic valve with pathology confined to the graft anastomosis, operative inspection and tissue acquisition, and integration of blood and tissue culture results with histopathology demonstrating organized thrombus without microorganisms. In this case, the aggregate findings were most consistent with anastomotic thrombosis, and multidisciplinary reassessment supported discontinuation of empiric antibiotics. However, because targeted serological and molecular testing for culture-negative endocarditis was not performed, culture-negative prosthetic graft infection could not be fully excluded, and the sterile designation remains presumptive. This distinction carries direct implications for antibiotic stewardship and the interpretation of similar future cases.
